# Supplementary material for: Electrostatic Collapse of Intrinsically Disordered Acid-Rich Protein Is Sensitive to Counterion Valency
Source: J Phys Chem Lett. 2025 Oct 14;16(42):10989–98. doi: 10.1021/acs.jpclett.5c02098 (PMC12557392; doi:10.1021/acs.jpclett.5c02098)
Supplement: Supplementary file 2 [file jz5c02098_si_002.pdf]

jz-2025-02098a.R1

Name: Peer Review Information for "Electrostatic Collapse of Intrinsically Disordered Acid-Rich Protein Is Sensitive to Counterion Valency"

First Round of Reviewer Comments

Reviewer: 1

Comments to the Author

Authors address a very important problem in the field of charge macromolecules. Intrinsically Disordered Proteins (IDPs) are rich in charge and a common assumption has been these charges are fully ionized. However charges may not be fully ionized due to the presence of counterions in the solution. To what extent do these counterions neutralize ionizable side chains ? This question has largely remained unanswered in IDPs even though it is a long-standing question in the field of polymer physics, particularly modeling uniformly charged polyelectrolytes. However some studies [Ruggeri et al, Pappu et al, Phillips et al] are emerging. In light of recent interest, the work is timely:

The manuscript clearly shows the importance of condensation by comparing changes in the chain dimension by varying the valency of the salt ions. Specifically they consider a highly negatively charged IDP called AGARP which collapses with addition of salt. However the degree of collapse is more enhanced with divalent salt than monovalent salt. This marked difference is seen using both FCS and SEC data. What is interesting is the collapse due to bivalent ions is more than the monovalent ions when considering the same ionic strength. The implication is bivalent ions are condensing more on the chain, effectively reducing the degree of ionization and hence the electrostatic repulsion. So the results of this experiment is important and will add to the emerging view on the importance of counterion condensation. I think the work very clearly shows there is condensation. However, there is one primary concern I have with the current interpretation of the measurement. Below I note down my concern and suggest edits which I believe will actually strengthen the paper and support their finding.

i) Authors rightfully make the case that divalent ions produce more condensation than monovalent ions. This makes sense and data clearly shows that. However they seem to imply monovalent ions do not cause condensation. I do not agree with this conclusion and suggest authors think carefully about this. Let's begin with their extracted Delta G^0 values (in Table 1) that ranges between 3-4 kT which does imply there is sufficient binding, albeit it is almost 1.5-2 times less than that of the divalent ions. Thus based on their own data I would argue even for monovalent ions there is some condensation.

ii) Based on the above, if we conclude there is condensation even for monovalent salt, it is actually consistent with previous work by Ruggeri et al Nature Nanotechnology, 102, 488, 2017. This work indeed reports an effective charge less than the structural charge for Starmaker, Prothymosin-alpha even for monovalent salts.

iii) Next, a recent work has shown again there is sufficient condensation even for monovalent salt (Phillips et al PNAS Nexus 3, 367, 2024). In this work authors developed a theory of condensation by coupling sequence and conformation for a general heteropolymer. When benchmarked against ProThymosin-Alpha data, this theory extracts condensation. Moreover, it shows models with condensation describes data better than model without condensation (see Figure 2). Interestingly, the typical parameters (p = 0.55) and assuming delta = 1 (a measure of dielectric mismatch which is expected to be close to that of water for highly expanded chains like AGARP) in equation 1 of this paper (ignoring f and alpha) would yield Delta G = 5 kT which is actually not too far from what authors got for monovalent ions. Thus once again I think authors finding for monovalent ion is infact showing there is some condensation and it is consistent with literature.

iv) So then the question arises: how to interpret their finding with Higgs-Joanny (HJ) using Debye-Huckel which seems to describe the results of the monovalent salt well ? I believe this is the basis of why authors think monovalent case has no condensation. Let me explain why this could be potentially problematic. I would note down few issues with HJ model. First off, it is a model that relies on composition and not exact sequence patterning. Moreover, equation 4 (in authors paper) is strictly valid for high salt regime although not explicitly stated in the original paper of HJ. The problem becomes evident when taking low salt limit (\lambda_D going to infinity) in equation 4. Note in this case, the exclude volume term actually diverges and it is not physical. Furthermore, note equation 4 has two terms:

one is polyampholytic attraction term that goes as $(f + g)^2 \lambda_d$ and then there is the polyelectrolytic repulsion term $(f-g)^2 \lambda_d^2$. The attraction term can significantly lower the repulsion. As a result this model can yield lower chain dimension than it actually is. In fact, a more rigorous theory has been developed by Huihui et al [JCP 149, 085101 2018] without such approximation of HJ. This theory actually takes into sequence patterning and not just composition (unlike HJ) and salt dependent dimension. So authors probably should use this more recent sequence dependent theory to analyze their data for monovalent salt. My guess is if this advanced theory is used, even for monovalent salt, authors will find the theory overestimates size. Now since this new theory (like HJ) also assumes full ionization and its failure to match data will imply there is condensation even for monovalent salt. In fact this has been done in the above mentioned paper by Phillips et al [PNAS Nexus] that overestimates size for ProT-alpha implying there is need to model condensation even for monovalent salt. If authors find this model difficult to apply they should probably still note these and mention the caveats of HJ theory.

In summary, I think the findings of the author that there is counterion condensation is very important and timely, but they need to reanalyze and revisit their statement about monovalent case has no condensation. I think much of their data actually shows there is also condensation for monovalent salt and this is consistent with the literature and if interpreted correctly will emphasize the role of condensation even more.

Reviewer: 2

Comments to the Author

The manuscript by Klepka et al describes how hydrodynamic dimensions of highly acid AGARP proteins are altered by the presence of monovalent and divalent cations. The authors use a combination of experimental (FCS, SEC) and computational (CG MD and direct Monte Carlo sampling) approaches to measure and model changes in protein chain dimensions. They find that while the effects of Na^+ and K^+ on AGARP Rh and Rg is well described by Debye-Huckel screening, additional ion binding interactions are needed to explain the protein chain compaction observed with Ca^{2+} and Mg^{2+} over a broadly similar range of overall ionic strength.

Interestingly, AGARP chain compaction induced by Ca^{2+} or Mg^{2+} ions is not associated with secondary structure formation (CD). A model in which cations bind to multiple sites on the AGARP protein chain is proposed, though lacks detail on what exactly these sites look like or how many of them there are on the protein chain. Experimental and computational work appears to have been caried out and analysed carefully. Overall, the results are interesting and in line with work on other highly negatively charged proteins such as OMM-64, starmaker, and ProTa. The manuscript is clearly written but could benefit from a broader discussion of what is currently known about how divalent cations can interact with and modulate (disordered) protein chains in solution.

Some thoughts and comments are listed below.

I relation to Figure 1 (FCS), the authors say that "The salt concentrations are chosen so that the ionic strength remains comparable across the panels (~150 mM for divalent salts, orange; ~450 mM for monovalent salts, blue)." In the legend for Figure 1, concentrations are given as "150 mM $CaCl_2$, 130 mM $MgCl_2$, 490 mM NaCl, and 320 mM KCl". While the trends in the data hold up, why were ionic strength-matched conditions not used? A colour key in Figure 1A would aid clarity.

It would be useful earlier on in the manuscript to state what the FCS and SEC derived Rh values/ranges are, instead of having them only presented in the plots in Figure 4, quite late on in the manuscript.

In Figure 2, labels of ionic strengths on the different SEC elution profiles would be helpful to orient the reader with the data presented.

Interaction strengths (ΔG) are described in kJ mol^{-1} (salt bridge) and kcal mol^{-1} (conversion from Kd). Consistent use of just one unit for ΔG would be helpful.

Can the authors speculate on the mode of divalent cation binding for AGARP? Does AGARP contain any established Ca^{2+} binding motifs e.g., EH-hand, Excalibur-like, or condensed-charge motifs (detailed in doi.org/10.3390/biom11081173)?

Is chain compaction caused by single Ca2+ ions bridging between two negatively charged sidechains? If so, can the number of binding sites in AGARP proteins be estimated from current data? How does it fit with the idea of "identical, non-interacting, entropically independent ion-binding sites", the number of Asp/Glu residues in the AGARP sequence, or other sequence patterning features?

Is Ca2+ ion-binding induced chain collapse associated with decreased overall chain charge? Has this been, or could this be, measured (e.g., doi.org/10.1016/j.celrep.2023.113375)?

Author's Response to Peer Review Comments:

August 30th, 2025

Responses to the Reviewers of "Electrostatic Collapse of Intrinsically Disordered Acid-Rich Protein Is Sensitive to Counterion Valency", Manuscript ID jz-2025-02098a

We are very grateful to the Reviewers for highlighting the shortcomings of the manuscript and providing suggestions on how to improve it. According to their valuable advice, we have taken steps to address these issues and to introduce merit corrections and amendments. Detailed responses to each of the concerns and a description of the changes of the text are set out below. We also attach the manuscript PDF and the supplementary information PDF with the tracked changes as "Supporting Information for Review Only".

Reviewer 1

Authors address a very important problem in the field of charge macromolecules. Intrinsically Disordered Proteins (IDPs) are rich in charge and a common assumption has been these charges are fully ionized. However charges may not be fully ionized due to the presence of counterions in the solution. To what extent do these counterions neutralize ionizable side chains? This question has largely remained unanswered in IDPs even though it is a longstanding question in the field of polymer physics, particularly modeling uniformly charged polyelectrolytes. However some studies [Ruggeri et al, Pappu et al, Phillips et al] are emerging. In light of recent interest, the work is timely:

The manuscript clearly shows the importance of condensation by comparing changes in the chain dimension by varying the valency of the salt ions. Specifically they consider a highly negatively charged IDP called AGARP which collapses with addition of salt. However the degree of collapse

is more enhanced with divalent salt than monovalent salt. This marked difference is seen using both FCS and SEC data. What is interesting is the collapse due to bivalent ions is more than the monovalent ions when considering the same ionic strength. The implication is bivalent ions are condensing more on the chain, effectively reducing the degree of ionization and hence the electrostatic repulsion. So the results of this experiment is important and will add to the emerging view on the importance of counterion condensation. I think the work very clearly shows there is condensation.

However, there is one primary concern I have with the current interpretation of the measurement. Below I note down my concern and suggest edits which I believe will actually strengthen the paper and support their finding.

i) Authors rightfully make the case that divalent ions produce more condensation than monovalent ions. This makes sense and data clearly shows that. However they seem to imply monovalent ions do not cause condensation. I do not agree with this conclusion and suggest authors think carefully about this. Let's begin with their extracted Delta G^0 values (in Table 1) that range between 3-4 kT which does imply there is sufficient binding, albeit it is almost 1.5-2 times less than that of the divalent ions. Thus based on their own data I would argue even for monovalent ions there is some condensation.

AUTHORS' REPLY: Thank you for bringing this to our attention, we have worked to improve the clarity of communication of the manuscript. We agree with the Reviewer's observations that the analysis using the apparent binding model indeed indicates a type of interaction between the protein and monovalent salts, that could be ascribed to a kind of weak counterion binding; the nature of this binding, however, cannot be determined from the binding constant alone and has to be compared against a reference model. In the case of a large molecule with multiple binding sites, comparing Delta G^0 with kT is a good rule of thumb, but more in depth analysis is needed to assess whether the bound ions simply form a diffuse cloud as predicted by Debye-Huckel theory or whether they exhibit an additional, stronger binding. According to the Reviewer's suggestion we have changed the text in the pages 14/15, as follows:

"Having established the difference in interaction strength between the cations and the protein, we focus on the nature of the interaction in more detail. Contrasting approaches, even in the case of a

monovalent salt, have been proposed, whereby some postulate to analyze the interaction as explicit condensation of ions on the protein ionized groups,[47] [BK1] *whilst others consider the interaction to be spatially diffuse.[21,48]* [BK2] *These different modes of interaction can be discerned by quantitative comparison of the experimental results with the predictions of the diffuse Debye-Hückel-like models, where deviations from these predictions would indicate the need to take additional binding mechanisms into account."*

ii) Based on the above, if we conclude there is condensation even for monovalent salt, it is actually consistent with previous work by Ruggeri et al Nature Nanotechnology, 102, 488, 2017. This work indeed reports an effective charge less than the structural charge for Starmaker, Prothymosin-alpha even for monovalent salts.

AUTHORS' REPLY: We agree that the condensation of the monovalent counterions in the vicinity of the protein can be described by the Debye-Huckel model. For example the properties of polyelectrolytes are influenced by a counterion cloud which moves together with the molecule. Here, again, the decrease in effective charge is not sufficient to tease out the mechanism of the binding to the protein – specific vs nonspecific, and has to be quantitatively compared against a non-specific, screening-induced condensation. According to the Reviewer's suggestion, we have added a more precise comment in page 19, as follows:

"The presented methodology for establishing additional binding effects is akin to that of Ruggeri et al.[48][BK1], whereby a model of the decrease in apparent charge was sought. Therein, the traditional screening model was sufficient to describe the interaction of counterions with prothymosin α, accounting for a 35% decrease in measured charge relative to structural charge. Likewise, in our work, we observe effects of diffuse interaction with monovalent salts. It should be underscored that this is not in contradiction with earlier analyses using the apparent binding model; rather, it represents a refinement that allows investigation of the mode of interaction, not merely the presence thereof. In contrast, Ca^{2+} and Mg^{2+} observations do not align with these simple predictions, and thus diffuse, Debye-like interaction alone is insufficient to explain their behavior."

iii) Next, a recent work has shown again there is sufficient condensation even for monovalent salt (Phillips et al PNAS Nexus 3, 367, 2024). In this work authors developed a theory of condensation by coupling sequence and conformation for a general heteropolymer. When benchmarked against ProThymosin-Alpha data, this theory extracts condensation. Moreover, it shows models with condensation describe data better than models without condensation (see Figure 2). Interestingly, the typical parameters (p = 0.55) and assuming delta = 1 (a measure of dielectric mismatch which is expected to be close to that of water for highly expanded chains like AGARP) in equation 1 of this paper (ignoring f and alpha) would yield Delta G = 5 kT which is actually not too far from what authors got for monovalent ions. Thus once again I think authors' finding for monovalent ions is in fact showing there is some condensation and it is consistent with literature.

AUTHORS' REPLY: Thank you for bringing this relevant work to our attention. We agree with the Reviewer that the condensation cannot be excluded. The model presented by Philips et al. requires estimating several parameters, with at least three needing to be fitted. While they demonstrate that their more complex model can more closely reproduce R_ee results, this outcome is expected due to the additional degrees of freedom introduced in the fitting procedure.

We agree that partial ionisation significantly influences the behaviour of any intrinsically disordered protein (IDP). However, the results of Philips et al. are not directly comparable to those presented in our study. The experimental validation included in their work is a charge measurement conducted at a very low ionic strength (approximately 1 mM). Additionally, the apparent charge measurements from Ruggeri et al. cited therein can be influenced by the counterion cloud that co-moves with the protein. As a result, comparing the theoretical prediction of charge of a (partially) ionised molecule alone with these experimental values of the charge of the molecule together with loosely bound counterions requires greater care.

We acknowledge that investigating the very low ionic strength regime is an exciting avenue for future research. However, we feel that at the present stage it falls outside the scope of this manuscript.

Following the Reviewer's comments, we have changed our manuscript by the above mentioned fragment on pages 14/15 with the reference to citation of Phillips at el.

iv) So then the question arises: how to interpret their finding with Higgs-Joanny (HJ) using Debye-Huckel which seems to describe the results of the monovalent salt well ? I believe this is the basis of why authors think monovalent case has no condensation. Let me explain why this could be potentially problematic. I would note down few issues with HJ model. First off, it is a model that relies on composition and not exact sequence patterning. Moreover, equation 4 (in authors paper) is strictly valid for high salt regime although not explicitly stated in the original paper of HJ. The problem becomes evident when taking low salt limit (\lambda_D going to infinity) in equation 4. Note in this case, the exclude volume term actually diverges and it is not physical.

AUTHORS' REPLY: We agree with the Reviewer that indeed the limit of \lambda_D \to \infty leads to nonphysical predictions. This is, however, not an issue in the presented data because of 10 mM Tris buffer present in all experiments, which leads to a minimal ionic strength of ca. 5 mM due to buffer ionization close to its pKa, corresponding to \lambda_D \approx 50 Å (comparable with the protein size). We agree that this might be large enough to make the deviations from idealised screening measurable, however this is beyond the scope of this publication and at the moment likely beyond the experimental resolution of the methods used. Note that FCS and SEC are in excellent agreement only down to around 50 mM NaCl. We would like to investigate the very low salt regime in our future work.

Following the Reviewer's comments, we have added a fragment on page 18 that clarifies the issue, as follows:

"Care is needed when using this approximation, as it hinges on large values of α; at the same time large α values correspond to large λ_D or small ionic strengths where the Higgs and Joanny approaches its limits of applicability due to divergence of interaction terms. These issues do not manifest in the data presented herein because of the minimal ionic strength of ca. half ionized 10 mM Tris present in all experiments. As such, λ_D never exceeds ~50 Å, which is comparable with the protein size, allowing for both the use of the Higgs and Joanny model and the convenient approximation. "

Furthermore, note equation 4 has two terms: one is polyampholytic attraction term that goes as (f + g)^2 \lambda_d and then there is the polyelectrolytic repulsion term (f-g)^2 \lambda_d^2. The attraction term can significantly lower the repulsion. As a result this model can yield a lower chain dimension than it actually is.

AUTHORS' REPLY: Thank you for raising this issue. The polyampholytic term is indeed important for the predictions presented. Its contribution is significant for higher ionic strengths (while for low

ionic strengths it is overwhelmed by the polyelectrolytic term). Below we include a graph comparing the experimental result with the HJ theory with and without the polyampholytic term. Note that we used the same values of the fitted excluded volume parameter, and some of the difference between the models can be compensated by adjusting it, thus the graph below should be used as a guide on the magnitude of the ampholytic contribution rather than true theory-experiment comparison.

Following the Reviewer's comment, we have prepared and added a Supplementary File S7, as shown below, as well as an additional comment on page18:

"Both the polyelectrolytic (self-repulsive) and the polyampholytic (self-attractive) interactions noticeably contribute to the theoretical amplitude of the electrostatic collapse. The magnitude of the ampholytic contribution can be examined visually in ***Figure S7****. "*

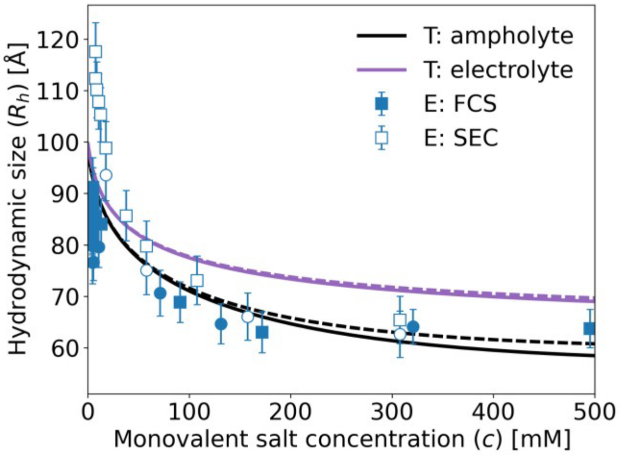

Supplementary Figure S7: Comparison of the theoretical model with both polyelectrolyte and polyampholyte contributions (black) with the theoretical model in which ampholitic contributions are disabled (purple). Excluded volume parameter: solid lines, 2.42; dashed lines, 2.87, the same as in the main text.

In fact, a more rigorous theory has been developed by Huihui et al [JCP 149, 085101 2018] without such an approximation of HJ. This theory actually takes into sequence patterning and not just composition (unlike HJ) and salt dependent dimension. So authors probably should use this more recent sequence dependent theory to analyze their data for monovalent salt.

AUTHORS' REPLY: We are very grateful to the Reviewer for turning our attention to this valuable paper. However, the theory proposed by Huihui et al 2018 includes both the electrostatic repulsion (polyelectrolytic behaviour) as well as electrostatic attraction (polyampholytic behaviour), thus will do little to alleviate any spurious collapse due to the overestimation of attractive interactions.

The second issue related to implementation of the theory proposed by Huihui et al. in our case is the requirement of fitting 4 parameters to a full atom molecular dynamics simulation at low salt concentration – this is beyond our current capabilities and likely beyond many groups' capabilities. The sequences of Huihui et al were significantly shorter (around 100 aa, compared to 506 aa for

AGARP) and simulations, even with an implicit solvent like the one proposed by Das & Pappu, are challenging and time consuming.
An alternative approach would be to fit the parameters to the measured experimental data. However, we believe this would lead to overfitting because of the limited number of features on the Rh(c) graph and the experimental precision.
Following the Reviewer's comments, we have included a fragment on page 4 with a reference to Huihui et al. 2018, as follows:
"A common assumption about polyelectrolyte or polyampholyte IDPs is that their charges are fully ionized. However, the extent to which the presence of counterions neutralizes ionizable amino acid side chains remains largely unanswered, despite a long-standing dispute in polymer physics regarding theories for modeling uniformly charged polyelectrolytes (DOI: 10.1021/acs.macromol.7b01929). Recently, some studies have addressed the coil-globule transition of IDPs depending on their charge patterning and ionic strength https://doi.org/10.1063/1.5037727."

My guess is if this advanced theory is used, even for monovalent salt, authors will find the theory overestimates size. Now since this new theory (like HJ) also assumes full ionization and its failure to match data will imply there is condensation even for monovalent salt. In fact this has been done in the above mentioned paper by Phillips et al [PNAS Nexus] that overestimates size for ProT-alpha implying there is need to model condensation even for monovalent salt. If authors find this model difficult to apply they should probably still note these and mention the caveats of HJ theory.

AUTHORS' REPLY: Following the Reviewer's suggestions, we have revised the main text to expand the discussion of existing research on monovalent salt condensation by the above mentioned fragment on pages 14/15 with the reference to citation of Phillips at el. The results of Philips et al. focussed on a regime of very low ionic strength; whether these predictions are directly applicable to a significantly different regime of moderate to high ionic strengths is still to be verified. Based on a good agreement of the Higgs and Joanny model, we postulate that the effect of partial ionisation on the hydrodynamic size, within the experimentally available regime, cannot be very large.

In summary, I think the findings of the author that there is counterion condensation is very important and timely, but they need to reanalyze and revisit their statement about monovalent case has no condensation. I think much of their data actually shows there is also condensation for monovalent salt and this is consistent with the literature and if interpreted correctly will emphasize the role of condensation even more.

AUTHORS' REPLY: We agree with the Reviewer and have rephrased some comments regarding the interpretation of the data. We agree that the condensation of the monovalent salts is present. However, we still believe that there is not enough data to prove that Debyelike, non-specific condensation cannot explain the variability observed with monovalent salts.

Reviewer 2:

The manuscript by Klepka et al describes how hydrodynamic dimensions of highly acid AGARP proteins are altered by the presence of monovalent and divalent cations. The authors use a combination of experimental (FCS, SEC) and computational (CG MD and direct Monte Carlo sampling) approaches to measure and model changes in protein chain dimensions. They find that while the effects of Na^+ and K^+ on AGARP Rh and Rg is well described by Debye-Huckel screening, additional ion binding interactions are needed to explain the protein chain compaction observed with Ca^{2+} and Mg^{2+} over a broadly similar range of overall ionic strength.

Interestingly, AGARP chain compaction induced by Ca^{2+} or Mg^{2+} ions is not associated with secondary structure formation (CD). A model in which cations bind to multiple sites on the AGARP protein chain is proposed, though lacks detail on what exactly these sites look like or how many of them there are on the protein chain. Experimental and computational work appears to have been carried out and analysed carefully. Overall, the results are interesting and in line with work on other highly negatively charged proteins such as OMM-64, starmaker, and ProTa. The manuscript is clearly written but could benefit from a broader discussion of what is currently known about how divalent cations can interact with and modulate (disordered) protein chains in solution.

Some thoughts and comments are listed below.

In relation to Figure 1 (FCS), the authors say that "The salt concentrations are chosen so that the ionic strength remains comparable across the panels (~150 mM for divalent salts, orange; ~450 mM for monovalent salts, blue)." In the legend for Figure 1, concentrations are given as "150 mM CaCl2, 130 mM MgCl2, 490 mM NaCl, and 320 mM KCl". While the trends in the data hold up, why were ionic strength-matched conditions not used? A colour key in Figure 1A would aid clarity.

AUTHORS' REPLY: We agree with the Reviewer that it would be better to have exactly equal divalent salt concentrations and the corresponding 3-fold higher monovalent salt concentrations. However, the FCS measurements were performed using a titration approach in protein sample droplets. The actual droplet volume was the result of both the titration with small amounts of salt stock solutions and the droplet evaporation during the time of the experiment that partially balances the dilution caused by titration. Therefore, the actual salt concentration in the droplet was calculated based on the actual droplet volume determined from the ratio of the number of molecules in the confocal volume before and after salt addition. Because the evaporation rates were not identical across salts, this made the exact ionic strength matching impossible. To highlight the differences in the autocorrelation curves of AGARP in the presence of mono- and divalent ions, we decided to present the data at comparable rather than strictly matched ionic strengths.

Following the Reviewer's comment, the explanation of how the actual salt concentration was determined using FCS in the sample droplets that underwent salt concentration- and

iondependent evaporation was added in the Supporting Information (page 5) and the reference to this explanation was added in the legend to Figure 1, as follows:

"For experimental details of actual salt concentration determination in the FCS experiment, see Supporting Information."

"For FCS, the actual concentrations of salts, Tris, and glycerol in the droplet were estimated based on the initial droplet volume, the number of moles of each component added, and the fitted number of fluorescent particles fitted from FCS to monitor water evaporation. Because the extent of water bound in ion hydration layers depends on the specific ions, evaporation rates were different across different salt solutions, this leading to variations in the measured actual salt concentrations., the number of moles of each component added, and the initial droplet volume."

A color code corresponding to salt concentrations has been added to Figure 1.

The Fig. 1 legend was supplemented by providing the explicit values of the ionic strengths.

It would be useful earlier on in the manuscript to state what the FCS and SEC derived Rh values/ranges are, instead of having them only presented in the plots in Figure 4, quite late on in the manuscript.

AUTHORS' REPLY: The manuscript has been revised in accordance with the Reviewer's recommendations by adding the following phrases in page 9:

"For both mono- and divalent salts, the R_h of AGARP decreases from ~80 Å at low salt concentrations to ~53 Å at high salt concentrations, with the onset of chain compaction occurring at lower salt concentrations for divalent ions than for monovalent ions."

"with the R_h of AGARP decreasing from ~113 Å at low salt to ~58 Å at high salt." (regarding SEC)

In Figure 2, labels of ionic strengths on the different SEC elution profiles would be helpful to orient the reader with the data presented.

AUTHORS' REPLY: Salt concentration labels were added to the SEC profiles in Figure 2.

Interaction strengths (ΔG) are described in kJ mol^{-1} (salt bridge) and kcal mol^{-1} (conversion from K_d). Consistent use of just one unit for ΔG would be helpful.

AUTHORS' REPLY: We are very grateful to the Reviewer for pointing this out. We have corrected the manuscript on page 14, and the necessary changes proved to be even more profound than just unit correction, as follows:

"The ΔG° values for the Ca^{2+} and Mg^{2+} cations are approximately twice as negative (ca. -4 kcal/mol) as those for the monovalent Na^+ and K^+ cations (ca. -2 kcal/mol).

Strikingly, the theoretical binding energy of a 3-Å single monovalent salt bridge in a water milieu (ε = 80) is approximately -1.4 kcal/mol, but it can achieve a magnitude 10-fold higher if buried inside a protein hydrophobic core (ε = 8). Depending on water accessibility and entropic contributions due to structure stabilization, the experimentally measured ΔG° of individual salt bridges ranged from -0.5 and -0.9

kcal/mol for lysozyme-antibody interactions[46] to ca. -3-5 kcal/mol for lysozyme folding[47]. Our ΔG° values fall in the range that corresponds to salt bridge formation. Considering that the binding involves water-accessible acidic residues, these results imply that the divalent cations are more tightly chelated, while the monovalent cations can only be loosely or transiently coordinated."

Can the authors speculate on the mode of divalent cation binding for AGARP? Does AGARP contain any established Ca^{2+} binding motifs e.g., EH-hand, Excalibur-like, or condensedcharge motifs (detailed in doi.org/10.3390/biom11081173)?

AUTHORS' REPLY: Thank you for raising these questions. AGARP does not contain any canonical EF-hand or Excalibur motifs in its sequence. However, its sequence includes short condensed-charge motifs (2-3 residues) and one longer, 7-residue-long acidic sequence. Such a regular distribution of Asp and Glu residues suggests that the predominant mode of divalent cation binding in AGARP could be chelation, which we describe in more detail in the next section. Following the Reviewer's questions, we have added a fragment of the discussion on pages 21/22 with seven new references and an additional Supporting Figure S8:

*"None of the canonical Ca^{2+}-binding motifs, such as EF-hand[52,53][BK1] or Excalibur[54,55][BK2], could be identified by Inter Pro[56][BK3] in the AGARP sequence. The protein exhibits an approximately uniform distribution of short condensed-charge motifs[57][BK4], with acidic residues primarily forming doublets or triplets, with one 7-residue-long condensed-charge motif (**Figure S8**). This observation leads us to postulate a chelation-like binding mode, reminiscent of how EDTA coordinates divalent cations[58,59][BK5] through four carboxylate groups from Asp or Glu side chains. Interestingly, the quantitative dependence of the collapse effect as a function of Ca^{2+} and Mg^{2+} activity is well explained by the apparent binding model, with the P-values from runs test from 0.26 to 0.96. This means that identical, non-interacting, entropically independent ion-binding sites on the protein molecule are a good first approximation of the binding mode. The millimolar affinity of AGARP to both Ca^{2+} and Mg^{2+} cations is much weaker than that of, e.g., mammalian intrinsically disordered extracellular matrix protein involved in biomineralization, osteopontin (OPN).[60][BK6] The affinity of OPN for Ca^{2+} was determined by isothermal titration calorimetry as K_d ~35 nM with the number of identical, non-interacting sites of ~10, while K_d for Mg^{2+} was ~2 μM with ~13 binding sites. OPN is less acidic (pI 4.46) and half as long (262 residues without the signal sequence) as AGARP (pI 3.94, 506 residues). However, its amino acid composition is more biased toward aspartic acid residues, with an Asp:Glu ratio of 1.30 vs. 0.88 for AGARP, which may explain the difference in the affinity.[57][BK7] Thus, AGARP can serve as a good model to test the polymeric behavior of highly charged IDPs at the limit of low affinity counterion interactions."*

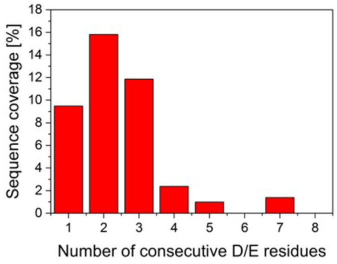

Figure S8. *Fraction of the AGARP sequence composed of consecutive D/E stretches of a given length. AGARP sequence is dominated by condensed-charge motifs composed of two or three acidic residues.*

Is chain compaction caused by single Ca^{2+} ions bridging between two negatively charged sidechains? If so, can the number of binding sites in AGARP proteins be estimated from current data?

AUTHORS' REPLY: Chain compaction could, in principle, arise from individual Ca^{2+} ions bridging between two or more negatively charged side chains located either close together or in distant positions in the sequence. Since the acidic amino acid residues in AGARP are grouped mainly in doublets and triplets that are almost evenly distributed in the sequence, we propose a chelation-like mechanism, reminiscent of how EDTA coordinates divalent cations, but much weaker. This mechanism aligns with the concept of identical, noninteracting, entropically independent ion-binding sites, since the sequence of AGARP does not contain any specific calcium-binding motifs. Only one larger condensed-charge motif is present in the sequence.
The exact number of binding sites cannot be estimated from current experimental data. This would require additional experiments involving counterion-specific fluorescent probes, which is a good question for future studies, but would exceed the scope of the present manuscript. At the moment, we can estimate that AGARP could bind up to several dozen Ca2+, which is consistent with the number of calcium ions bound to the OMM-64 protein (10.1016/j.bbapap.2017.08.019). Like AGARP, OMM-64 has its Asp and Glu residues evenly distributed along the sequence, with a similar total number of negatively charged amino acid residues (213), and has been shown to bind 61 ± 7 Ca^{2+} ions, which is well within the expected range based on the chelation-like mechanism. However, we would like to avoid speculations without having experimental evidence. Looking ahead, we welcome the opportunity to further elaborate the mechanisms of the binding and the collapse.
Following the Reviewer's comments, we have added a new discussion fragment on page 24, as follows:
"The chain compaction caused by single Ca^{2+} ions can bridge two or more negatively charged AGARP side chains, resembling the chelation of cations by EDTA or EGTA, but with much weaker strength due to non-optimal geometry and the need of the protein chain reconfiguration. Moreover, we have recently observed the aggregation of AGARP molecules at a submicromolar concentration upon contact with Ca^{2+} ions during diffusion through a confocal volume, leading to the deposition of amorphous calcium carbonate,[32] which means that the overall protein charge had to be neutralized by counterions. For this study, however, we excluded all fluorescence count rate spikes reflecting the aggregation events from the FCS analysis to obtain the hydrodynamic dimensions of solely monomeric protein chains. Thus, inferring the total neutralization of the protein charge by the direct counterion binding to the chain at the titration saturation plateau would not be entirely justified here. On the other hand, the need to use a low protein concentration to prevent the aggregation hinders the ability to determine the exact number of binding sites using ITC or to measure the actual charge based on the z-potential.[61]"

How does it fit with the idea of "identical, non-interacting, entropically independent ionbinding sites", the number of Asp/Glu residues in the AGARP sequence, or other sequence patterning features?
AUTHORS' REPLY: This issue was discussed above when the possible mode of divalent cation binding by AGARP was commented on (a new fragment on pages 21/22 in the manuscript).

Is Ca2+ ion-binding induced chain collapse associated with decreased overall chain charge? Has this been, or could this be, measured (e.g., doi.org/10.1016/j.celrep.2023.113375)?

AUTHORS' REPLY: This question requires a nuanced answer – indeed the reduction of the apparent chain charge has already been directly observed for another protein, prothymosin alpha, in response to monovalent cation interaction [Rugerri et al.]. This charge reduction is, on the other hand, well explained by simple electrostatic theory akin to Debye-Huckel (more details in the cited publication). Other authors claim that counterions can also influence the intrinsic charge of the chain itself by direct condensation [Philips et al] although this has not been satisfactorily confirmed in experiment or numerical simulation.
Based on the discrepancy between the interaction strength with monovalent and divalent salts we postulate that some Ca2+ ions are tightly bound, directly to the chain (as pictured in the toy model), thus decreasing the intrinsic chain charge.
A new discussion fragment on page 24 that addresses the Reviewer's question was cited above when commenting on the possible number of Ca2+ binding sites.

All the above and other minor changes introduced into the main manuscript text and supporting information are displayed in their PDF versions.

Source data for the figures are deposited at: https://doi.org/10.18150/7DLIT3

Once again, we would like to thank the Reviewers for their valuable advice on how to improve our work.

Anna Niedźwiecka, PhD
